# Nitrate Is an Environmental Cue in the Gut for Salmonella enterica Serovar Typhimurium Biofilm Dispersal through Curli Repression and Flagellum Activation via Cyclic-di-GMP Signaling

**DOI:** 10.1128/mbio.02886-21

**Published:** 2022-02-08

**Authors:** Amanda L. Miller, Lauren K. Nicastro, Shingo Bessho, Kaitlyn Grando, Aaron P. White, Yi Zhang, Gillian Queisser, Bettina A. Buttaro, Çagla Tükel

**Affiliations:** a Center for Microbiology and Immunology, Lewis Katz School of Medicine, Temple University, Philadelphia, Pennsylvania, USA; b Vaccine and Infectious Disease Organization-International Vaccine Centre, Saskatoon, Saskatchewan, Canada; c Fels Institute for Cancer Research and Molecular Biology, Lewis Katz School of Medicine, Temple University, Philadelphia, Pennsylvania, USA; d Department of Mathematics, College of Science and Technology, Temple University, Philadelphia, Pennsylvania, USA; e Thrombosis Research Center, Lewis Katz School of Medicine, Temple University, Philadelphia, Pennsylvania, USA; Washington University School of Medicine

**Keywords:** *Salmonella*, biofilms, c-di-GMP, curli, cyclic GMP, flagella, gut inflammation, nitrate

## Abstract

Curli, a major component of the bacterial biofilms in the intestinal tract, activates pattern recognition receptors and triggers joint inflammation after infection with Salmonella enterica serovar Typhimurium. The factors that allow *S.* Typhimurium to disperse from biofilms and invade the epithelium to establish a successful infection during acute inflammation remain unknown. Here, we studied *S.* Typhimurium biofilms *in vitro* and *in vivo* to understand how the inflammatory environment regulates the switch between multicellular and motile *S.* Typhimurium in the gut. We discovered that nitrate generated by the host is an environmental cue that induces *S.* Typhimurium to disperse from the biofilm. Nitrate represses production of an important biofilm component, curli, and activates flagella via the modulation of intracellular cyclic-di-GMP levels. We conclude that nitrate plays a central role in pathogen fitness by regulating the sessile-to-motile lifestyle switch during infection.

## INTRODUCTION

Nontyphoidal Salmonella enterica serovar Typhimurium (*S.* Typhimurium) causes gastroenteritis, an inflammatory diarrhea accompanied by fever, nausea, and abdominal cramping in immunocompetent individuals. *S.* Typhimurium causes an estimated 93.5 million infections per year (1). *S.* Typhimurium cycles between a sessile, multicellular form and a planktonic cell form, allowing it to successfully survive in the environment and thrive in the host. The multicellular biofilm lifestyle is thought to aid in the transmission of the pathogen as it helps bacteria to persist in the presence of disinfectants, antibiotics, or chemical, physical, and mechanical stresses. Once *S.* Typhimurium is ingested by the host, the pathogen travels through the gastrointestinal tract. Flagella enable the planktonic bacteria to swim toward the intestinal epithelial cells, and a type III secretion system (T3SS) facilitates invasion of the epithelium (2–4). After crossing the intestinal barrier, intestinal inflammation initiated by the detection of the pathogen via pattern recognition receptors leads to an inflammatory response that facilitates *S.* Typhimurium growth in the lumen of the intestinal tract (5, 6). Recently, we showed that *S.* Typhimurium produces the protein curli, which is a major component of the biofilm matrix, in the intestinal tract during infection (7, 8). This suggests that two populations are present in the intestinal tract: (i) cells that form biofilms that are adapted for persistence and survival and (ii) planktonic cells that express T3SS proteins as well as flagella and are adapted for virulence (2, 7, 9). The signaling mechanisms that drive virulence and manipulate interactions with the host remain unknown.

The extracellular matrix of the *S.* Typhimurium biofilm is composed of amyloid curli, cellulose, DNA, and BapA (10). Curli are the main proteinaceous component, accounting for up to 85% of the extracellular matrix, and are responsible for the development of the overall biofilm architecture (11–13). Two divergently transcribed operons, *csgBAC* and *csgDEFG*, encode the curli subunits and the machinery that controls their export (9, 14). CsgD is a key regulator of the *csg* gene cluster and also regulates many other genes involved in biofilm formation. CsgD expression is regulated by environmental stimuli such as temperature, growth phase, and the levels of second messenger cyclic-di-GMP (c-di-GMP) (15–17). High intracellular levels of c-di-GMP promote CsgD activation, leading to the activation of downstream genes involved in biofilm formation. CsgD activates the expression of *csgBAC*, leading to the production of the major and minor subunits of curli and expression of AdrA, which further transcriptionally increases cellulose synthase and cellulose biosynthesis (11, 18). Thus, c-di-GMP stimulates the production of matrix components such as cellulose and curli, promoting biofilm formation, and inhibits motility (17, 19, 20). Conversely, decreased levels of c-di-GMP lead to decreased biofilm formation and increased motility *in vitro* (12, 21).

Various pathogens form biofilms *in vivo* as a strategy for immune system evasion and for increased persistence. Multicellular aggregates that express curli have increased resistance to complement-mediated killing (13). Conversely, curli are recognized by the innate immune cells within in the intestinal tract via the complex containing CD14 and Toll-like receptors TLR2 and TLR1 (22–25). Epithelial cells directly respond to curli-expressing *S.* Typhimurium and limit bacterial translocation during infection via TLR2-mediated immune responses (26).

High levels of nitrate and tetrathionate as well as other metabolites that are generated in the gastrointestinal mucosa during inflammation serve as a chemoattractant for *S.* Typhimurium (27–29). The proteins encoded by *S.* Typhimurium chemotaxis genes *tsr* and *aer* cause migration toward areas of increased inflammation and consequently higher concentrations of nitrate and tetrathionate, respectively (15). Recently, the Keio collection of single-gene deletions was used to identify Escherichia coli mutants with defective amyloid production (16). Mutations in *narQ*, which encodes the primary sensor of nitrate, resulted in decreased CsgD and CsgA levels when bacteria were grown in biofilm-inducing conditions, indicative of a possible link between the ability to sense nitrate concentrations and curli production (16). Here, we investigated whether nitrate could serve as an *in vivo* signal for *S.* Typhimurium. Indeed, we found that nitrate regulates biofilm formation and potentially serves as a switch between a sessile and motile lifestyle.

## RESULTS

### Nitrate attenuates *S.* Typhimurium production of curli and cellulose during biofilm formation.

**FIG 1:**
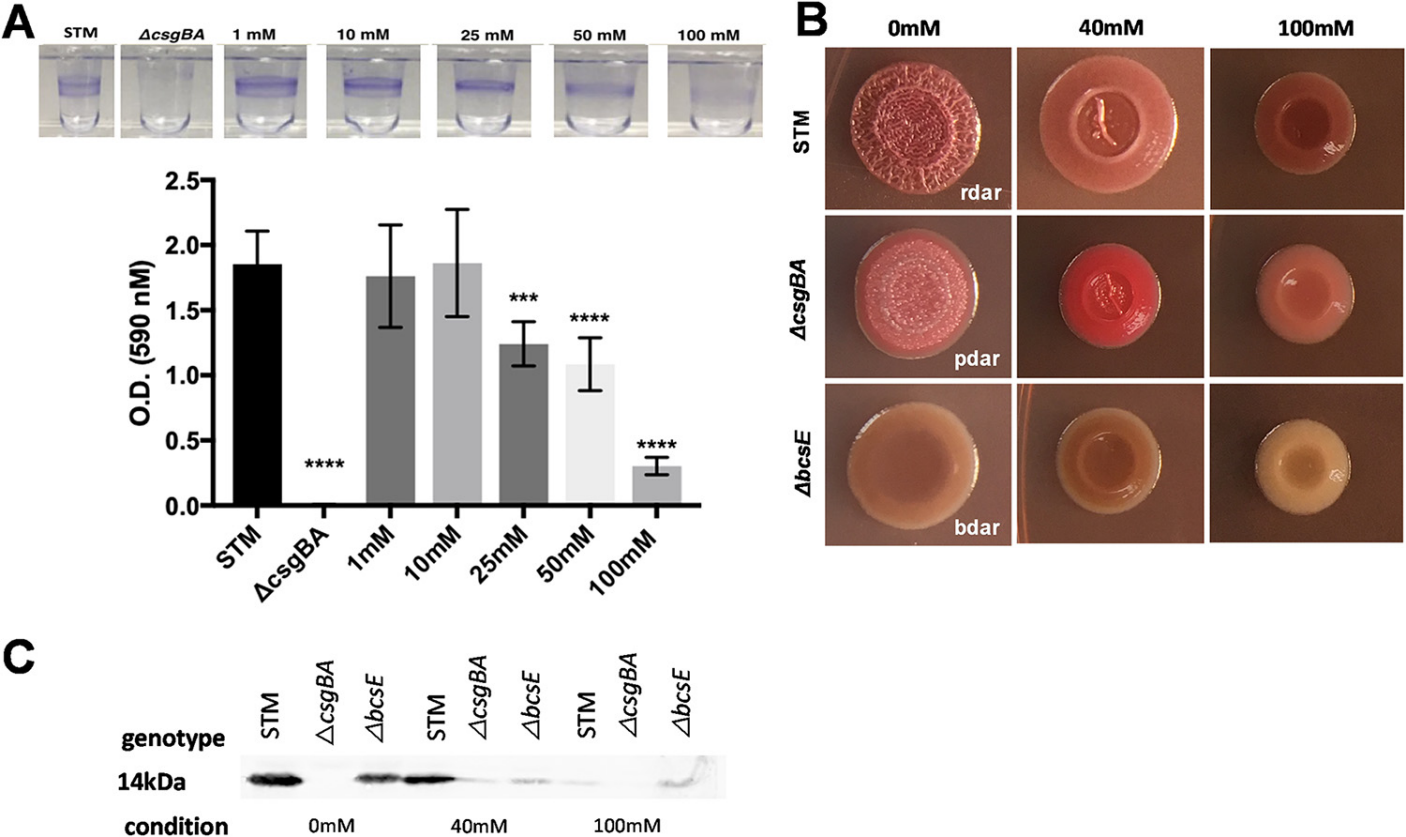
Addition of nitrate alters the biomass and expression of curli and cellulose in *S.* Typhimurium biofilms. (A) Biofilm pellicles of wild-type *S.* Typhimurium grown with or without NaNO_3_ for 72 h at 28°C were stained with a crystal violet assay. (B) Colony biofilms of wild-type *S.* Typhimurium, *csgBA* mutant, and *bcsE* mutant spotted onto Congo red/Coomassie blue indicator plates supplemented with 0 mM, 40 mM, or 100 mM NaNO_3_. (C) Western blot of extracts of wild-type *S.* Typhimurium, *csgBA* mutant, and *bcsE* mutant grown in 0 mM, 40 mM, or 100 mM NaNO_3_ for CsgA.

*S.* Typhimurium forms biofilms on both biotic and abiotic surfaces. *S.* Typhimurium forms a pellicle biofilm at the air-liquid interface in liquid medium, whereas it forms a colony biofilm on solid media. To investigate the effect of nitrate on *S.* Typhimurium biofilms, we characterized *S.* Typhimurium biofilms grown in the presence of sodium nitrate (NaNO_3_). Briefly, overnight cultures were grown statically in no-salt Luria-Bertani (LB) broth supplemented with increasing concentrations of NaNO_3_ for 72 h at 28°C. Incubation of *S.* Typhimurium with 25 mM, 50 mM, or 100 mM NaNO_3_ visibly reduced pellicle formation at the air-liquid interface compared to cultures grown in the absence of NaNO_3_ (Fig. 1A). No pellicle was observed in cultures of the isogenic curli mutant (*csgBA*), used as a negative control. When the surface-attached pellicle was quantified using crystal violet as a proxy for biofilm mass, we determined that 25 mM, 50 mM, and 100 mM NaNO_3_ significantly decreased the overall biomass of the biofilm compared to cultures grown without NaNO_3_ (Fig. 1A). To ensure that this reduction was due to nitrate and not sodium toxicity, we repeated the same assay using potassium nitrate (KNO_3_) and saw a similar trend with significant decreases of the overall biomass of the biofilm at 25 mM, 50 mM, and 100 mM concentrations of KNO_3_ (Fig. S1). In subsequent experiments, we used 40 mM and 100 mM NaNO_3_ concentrations, as 40 mM has been reported to be the physiological concentration of nitrate during *S.* Typhimurium infection and inflammation. Concentrations above 40 mM are expected in inflamed areas close to the epithelium (30–33).

10.1128/mBio.02886-21.1 FIG S1 Potassium nitrate significantly decreases biofilm mass. Wild-type *S.* Typhimurium grown in the presence or absence of KNO_3_. Biofilms were quantified using Crystal violet staining. ****, *P* < 0.0001. Download FIG S1, TIF file, 0.4 MB. Copyright © 2022 Miller et al. 2022 Miller et al. https://creativecommons.org/licenses/by/4.0/ This content is distributed under the terms of the Creative Commons Attribution 4.0 International license.

Next, we examined the morphologies of colony biofilms of wild-type *S.* Typhimurium, its curli mutant, and its cellulose (*bcsE*) mutant in the presence of NaNO_3_. Normally, when grown on yeast extract Casamino Acid (YESCA) supplemented with Congo red and Coomassie blue, wild-type *S.* Typhimurium has a red, dry, and rough morphotype, whereas the curli mutant has a brown, dry, and rough morphotype; the cellulose mutant has a pink, dry, and rough morphotype; and the curli/cellulose double mutant has a smooth and white morphotype (14, 34–39). We observed a change in the morphotypes of all three strains when grown on the indicator plates supplemented with 40 mM or 100 mM NaNO_3_. The wild type and *csgBA* mutant lost their rough textures and became smoother in appearance, whereas the *bcsE* mutant had a loss in color (Fig. 1B). There was also a significant decrease in the average size of the colonies at 100 mM concentration of nitrate (see Fig. S2 in the supplemental material). These morphotype changes suggest that the presence of nitrate led to decreases in curli production during the formation of the biofilm.

10.1128/mBio.02886-21.2 FIG S2 Nitrate decreased colony size in all strains. Wild-type *S*. Typhimurium, *csgBA* mutant, and *bcsE* mutant were grown in the absence or presence of nitrate. The diameter of each colony was measured. ****, *P* < 0.0001. Download FIG S2, TIF file, 0.4 MB. Copyright © 2022 Miller et al. 2022 Miller et al. https://creativecommons.org/licenses/by/4.0/ This content is distributed under the terms of the Creative Commons Attribution 4.0 International license.

To confirm the effect of nitrate on curli production, we measured CsgA protein levels in whole-cell extracts obtained from colony biofilms. Briefly, single colonies were scraped from dye-free YESCA and resuspended in PBS, and their optical densities (OD) were normalized. Hexafluoroisopropanol (HFIP) was used to depolymerize curli into CsgA monomers. Western blot analysis of the protein extracts showed that CsgA protein levels were decreased in both the wild-type and *bcsE* mutant strains grown in the presence of nitrate, with the most evident reduction in band size occurring at 100 mM (Fig. 1C). No bands corresponding to CsgA were observed in the *csgBA* mutant extracts. Overall, these results suggest that nitrate acts as a signal that causes Salmonella to decrease the expression of a major biofilm component, curli.

### Nitrate decreases the levels of the second messenger c-di-GMP, leading to a reduction in biofilm formation and an increase in motility.

**FIG 2:**
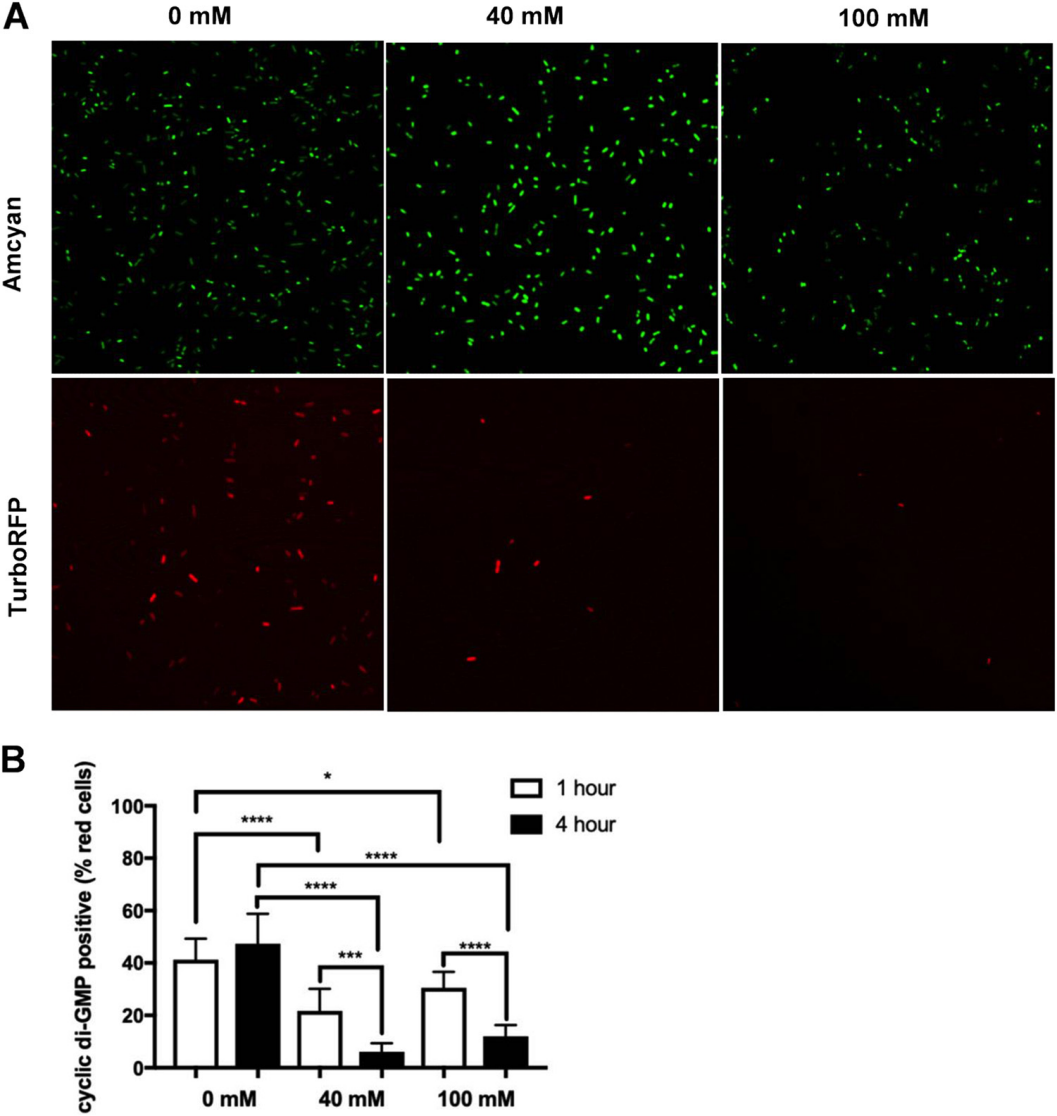
Nitrate decreases the levels of second messenger c-di-GMP in Salmonella. (A) Images of wild-type *S.* Typhimurium transformed with pMMB-Gm-Bc3-5AAV plasmid were grown overnight under biofilm-inducing conditions then were treated with NaNO_3_ for 1 to 4 h. Bacteria were imaged using a TC5 confocal microscope at 100× oil magnification. (B) The percentage of c-di-GMP-positive cells determined as the number of TurboRFP-positive cells divided by the number of Amcyan-positive cells for each individual field × 100. ****, *P* < 0.0001; ***, *P* = 0.0003.

One of the signals that regulates biofilm formation is the second messenger c-di-GMP. c-di-GMP also regulates other cellular functions, including cell cycle progression, virulence, and motility. Since biofilm formation and motility are inversely regulated by c-di-GMP levels (17, 40–43), we tested whether c-di-GMP levels were modulated by nitrate under biofilm-inducing conditions. To do this, we utilized a c-di-GMP reporter plasmid, pMMB-Gm-Bc3-5 AAV (pFY_4950) (44). The bacteria that express the constitutively active plasmid fluoresce green, but when intracellular levels of c-di-GMP increase, TurboRFP is activated and the bacteria fluoresce red. *S.* Typhimurium containing the pFY_4950 plasmid was grown overnight in liquid culture under biofilm-inducing conditions before cells were treated with 40 mM or 100 mM NaNO_3_. Bacteria were collected 1 h and 4 h posttreatment and imaged using confocal microscopy (Fig. 2A). Green cells as a count of total cells and red cells (c-di-GMP positive) were counted using ImageJ, and the ratio of red to green cells was calculated (shown as a percentage of red cells). Significantly fewer red cells were seen 4 h after cells were treated with 40 mM or 100 mM NaNO_3_ compared to their 1-h counterparts or to cells not treated with NaNO_3_ (Fig. 2B), indicating that nitrate exposure led to a decrease in the intracellular levels of c-di-GMP.

### Nitrate enhances migration and reduces aggregation in biofilm.

**FIG 3:**
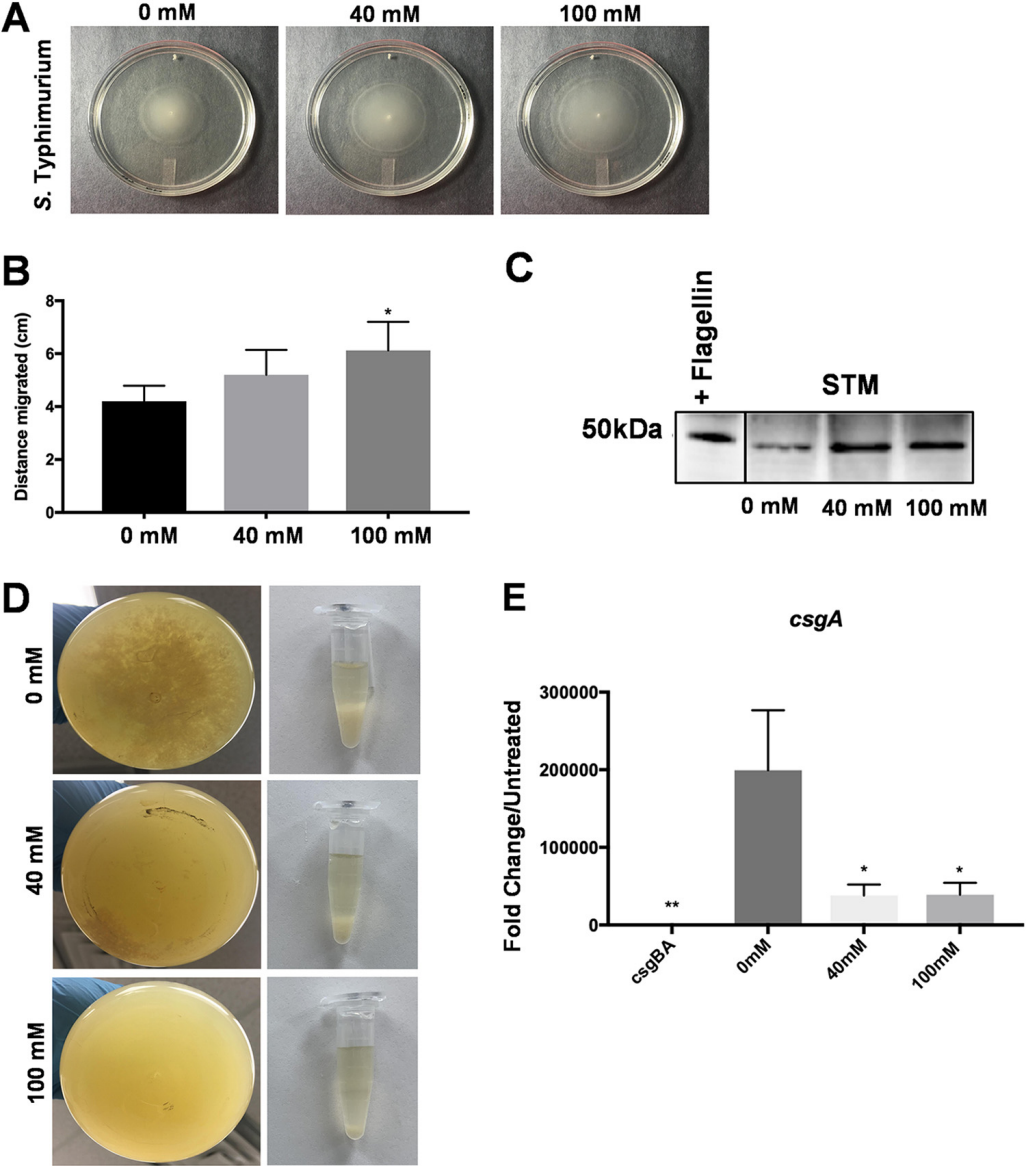
Addition of nitrate under biofilm-inducing conditions enhances motility of Salmonella. (A) Single colonies of wild-type *S.* Typhimurium inoculated in a 0.03% agar plate with or without 40 or 100 mM NaNO_3_. Photographs were taken after incubation overnight at 28°C. Bacterial motility was determined by measuring the halo created by the bacteria as they swam. (B) Distance migrated by *S.* Typhimurium on plates with and without NaNO_3_. (C) Western blot of extracts of *S.* Typhimurium incubated with or without NaNO_3_ for flagellin. (D) Batch cultures of wild-type *S.* Typhimurium were grown shaking at 28°C for 72 h to induce biofilm production. NaNO_3_ was added to each culture and shaken for an additional 4 h. (Left) Photographs demonstrating decreases in aggregation in the bottom of the flasks as NaNO_3_ concentration increases. (Right) Sedimentation of aggregates collected from the flask and allowed to settle in Eppendorf tubes. (E) Fold change in *csgA* expression in biofilm-former populations from cultures supplemented with NaNO_3_ determined by RT-PCR relative to the curli mutant.

Since c-di-GMP inversely regulates biofilm formation and motility *in vitro* (12), we next investigated if the presence of nitrate increased bacterial motility. To test this, we measured the motility of *S.* Typhimurium on soft-agar motility plates supplemented with nitrate and incubated at 28°C or 37°C. Lower levels of motility were observed at every concentration of NaNO_3_ tested under biofilm-inducing conditions at 28°C compared to the invasive condition at 37°C (Fig. S3). Plates incubated at 37°C had halos about three times as large as those incubated at 28°C. Moreover, at 28°C, the bacteria traveled significantly further in the presence of 100 mM NaNO_3_ than in the absence of nitrate over the same amount of time (Fig. 3A and B). When wild-type *S.* Typhimurium bacterial extracts were subjected to Western blot analysis, we detected higher levels of flagellar proteins in bacteria exposed to 40 mM and 100 mM NaNO_3_ than in extracts of bacteria not exposed to nitrate, with the largest amount of protein detected in 100 mM NaNO_3_ (Fig. 3C).

10.1128/mBio.02886-21.3 FIG S3 Nitrate increased motility and halo formation in biofilm inducing conditions. A single colony of wild-type S. Typhimurium was inoculated in a 0.03% agar plate, and the motility assay was performed at 28°C (biofilm-inducing conditions) or 37°C (invasive conditions) in the presence or absence of nitrate. The diameter of each halo was measured. Download FIG S3, TIF file, 0.6 MB. Copyright © 2022 Miller et al. 2022 Miller et al. https://creativecommons.org/licenses/by/4.0/ This content is distributed under the terms of the Creative Commons Attribution 4.0 International license.

To confirm that this was due to a change in migration ability rather than to altered growth, we repeated the motility assay with two nitrate reductase mutants, Δ*narX* and Δ*narL* strains, and the Δ*tsr* chemotaxis mutant. There were no differences in motility of the nitrate reductase mutants in the presence and absence of nitrate; however, the *tsr* mutant did not migrate even in the presence of the highest concentrations of nitrate (Fig. S4). Overall, these results suggest that nitrate is detected by bacteria present in biofilms, and this induces the bacteria to become planktonic and switch on their flagellar motility.

10.1128/mBio.02886-21.4 FIG S4 Increased motility of *S.* Typhimurium is a result of chemotaxis, not altered growth. Single colonies of two nitrate reductase mutants, *narX* and *narL*, and one chemotaxis mutant, *tsr*, were inoculated into a 0.03% agar plate in the absence or presence of nitrate. The diameter of each halo was measured. ***, *P* < 0.001. Download FIG S4, TIF file, 0.5 MB. Copyright © 2022 Miller et al. 2022 Miller et al. https://creativecommons.org/licenses/by/4.0/ This content is distributed under the terms of the Creative Commons Attribution 4.0 International license.

Biofilms are composed of a heterogeneous population of biofilm formers and a population of more metabolically active and motile cells that leave the biofilms to initiate other processes, such as seeding of a new biofilm or invasion (9). Bacterial cells can switch between these two states in response to the signals received from the environment. To elucidate nitrate’s role in this switch, we began by quantifying curli and flagellar expression within these two populations. *S.* Typhimurium was grown under biofilm-inducing conditions in batch culture for 24 h, and then NaNO_3_ was added or not and cultures were shaken for an additional 1 to 4 h. There was a noticeable decrease in the visible amount of aggregation and sedimentation in the cultures treated with nitrate (Fig. 3D).

The two populations were separated using low-speed centrifugation as previously described (9). This low-speed centrifugation pellets the biofilm formers while the planktonic population remains in the supernatant. The biofilm-forming population was isolated and analyzed for expression of *csgA* (Data Set S1). There was a significant decrease in *csgA* expression when the biofilm cells were treated with 40 mM or 100 mM NaNO_3_ (Fig. 3E). Taken together, these data show that biofilm formers shift from a sessile state to a more planktonic state in the presence of nitrate.

10.1128/mBio.02886-21.7 DATA SET S1 Raw data for expression of *csgA*. After bacteria were grown under biofilm-inducing conditions with the addition of 0mM, 40mM, or 100mM NaNO_3_, biofilm aggregates and planktonic bacteria were separated using low-speed centrifugation. Expression of *csgA* was determined in the aggregates. Download Data Set S1, XLS file, 0.2 MB. Copyright © 2022 Miller et al. 2022 Miller et al. https://creativecommons.org/licenses/by/4.0/ This content is distributed under the terms of the Creative Commons Attribution 4.0 International license.

### Addition of nitrate alters the architecture of the mature biofilm.

**FIG 4:**
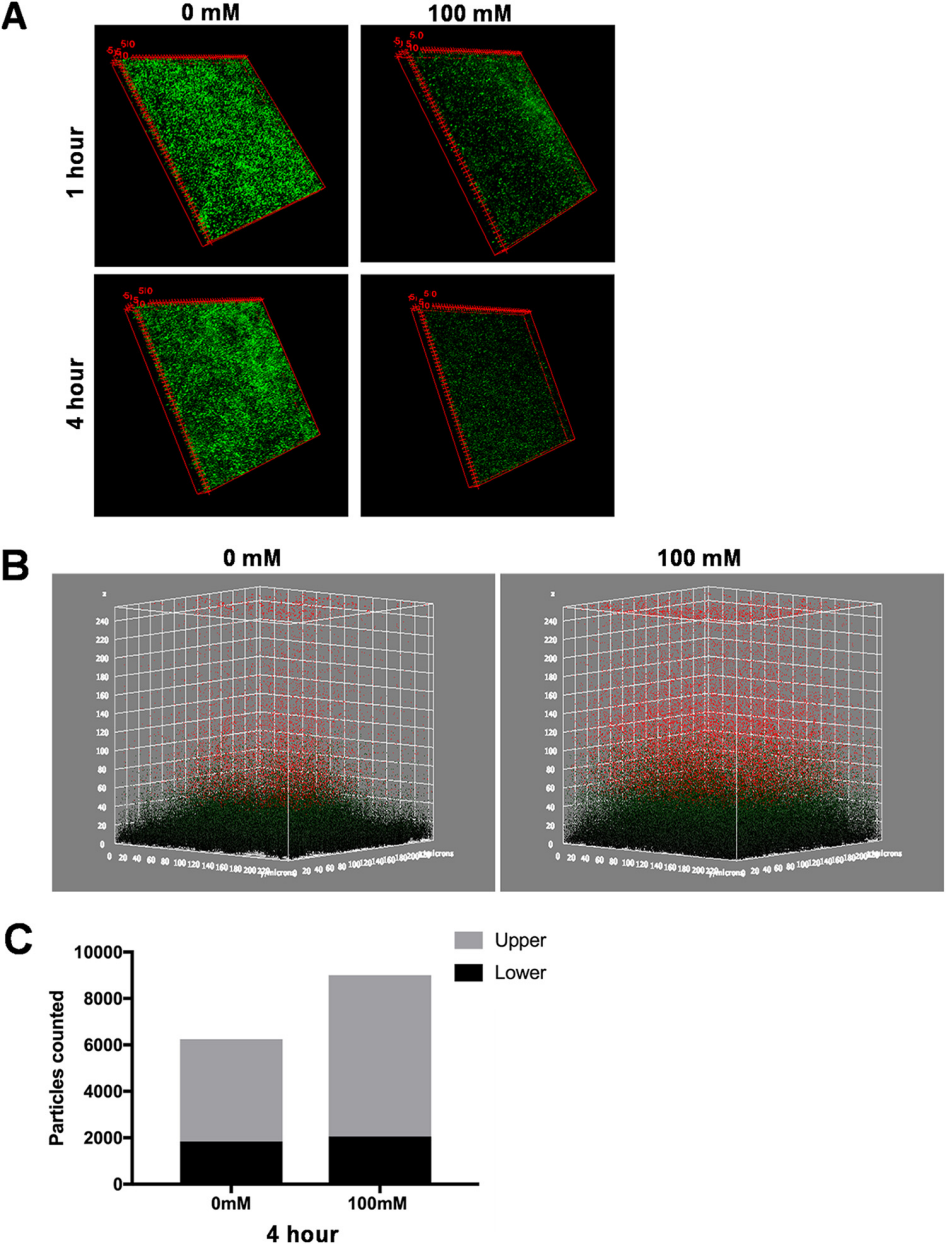
Addition of nitrate alters the architecture of the mature biofilm. (A) 3D views of untreated and nitrate-treated wild-type *S.* Typhimurium biofilms. Biofilms were grown on round coverslips for 72 h at 28°C. The mature biofilms were washed and treated with 100 mM NaNO_3_ for 1 or 4 h before staining with SYTO 9 (green) and imaged using confocal microscopy (63× oil magnification). (B) 3D surface plots were created of the 4-h time point, forming two distinct layers, a less compact upper layer (red particles) and a compact lower layer (green particles). (C) Quantification of 3D surface plots showing an increase in the number of particles in the upper layer (red particles).

To test the effect of nitrate on the architecture of mature established biofilms, *S.* Typhimurium biofilms were grown for 72 h on glass coverslips in a 24-well plate under biofilm-inducing conditions. Mature biofilms were treated with 0 mM and 100 mM NaNO_3_ for 1 to 4 h and then imaged using confocal microscopy. In three-dimensional (3D) views, those biofilms treated with nitrate were sparser than untreated biofilms (Fig. 4A). We used a 3D surface plot for a more in-depth look at the architecture of the biofilms. Biofilms were considerably less compact in the presence of nitrate than in its absence, and the nitrate-treated biofilms appeared to separate into two distinct regions, a compact lower layer (green) and a loose upper layer (red) (Fig. 4B). Furthermore, there were more particles in the upper layer of the nitrate-treated biofilms than those not treated with nitrate, although the thicknesses of the lower layers remained similar (Fig. 4C). Furthermore, supernatant was collected from nitrate-treated biofilms at 1 and 4 h. These samples were diluted and plated for bacterial enumeration. Significantly more bacteria were found in the supernatant of nitrate-treated biofilms at 4 h than 1 h. There was also a significant difference between the groups at 4 h, with higher levels of bacteria at 40 mM and 100 mM than at 0 mM (Fig. S5). Taken together, these data suggest the bacteria in the exterior regions of the biofilm sense and chemotax toward the nitrate that is in the environment, initiating the dispersal of the biofilm.

10.1128/mBio.02886-21.5 FIG S5 Nitrate increased bacterial dispersion from mature biofilms. Mature wild-type *S.* Typhimurium biofilms were treated with or without nitrate. Supernatant was collected at 1- and 4-h time points, diluted, and plated for bacterial enumeration. *, *P* < 0.005. Download FIG S5, TIF file, 0.3 MB. Copyright © 2022 Miller et al. 2022 Miller et al. https://creativecommons.org/licenses/by/4.0/ This content is distributed under the terms of the Creative Commons Attribution 4.0 International license.

### Nitrate disrupts integrity of mature biofilms.

**FIG 5:**
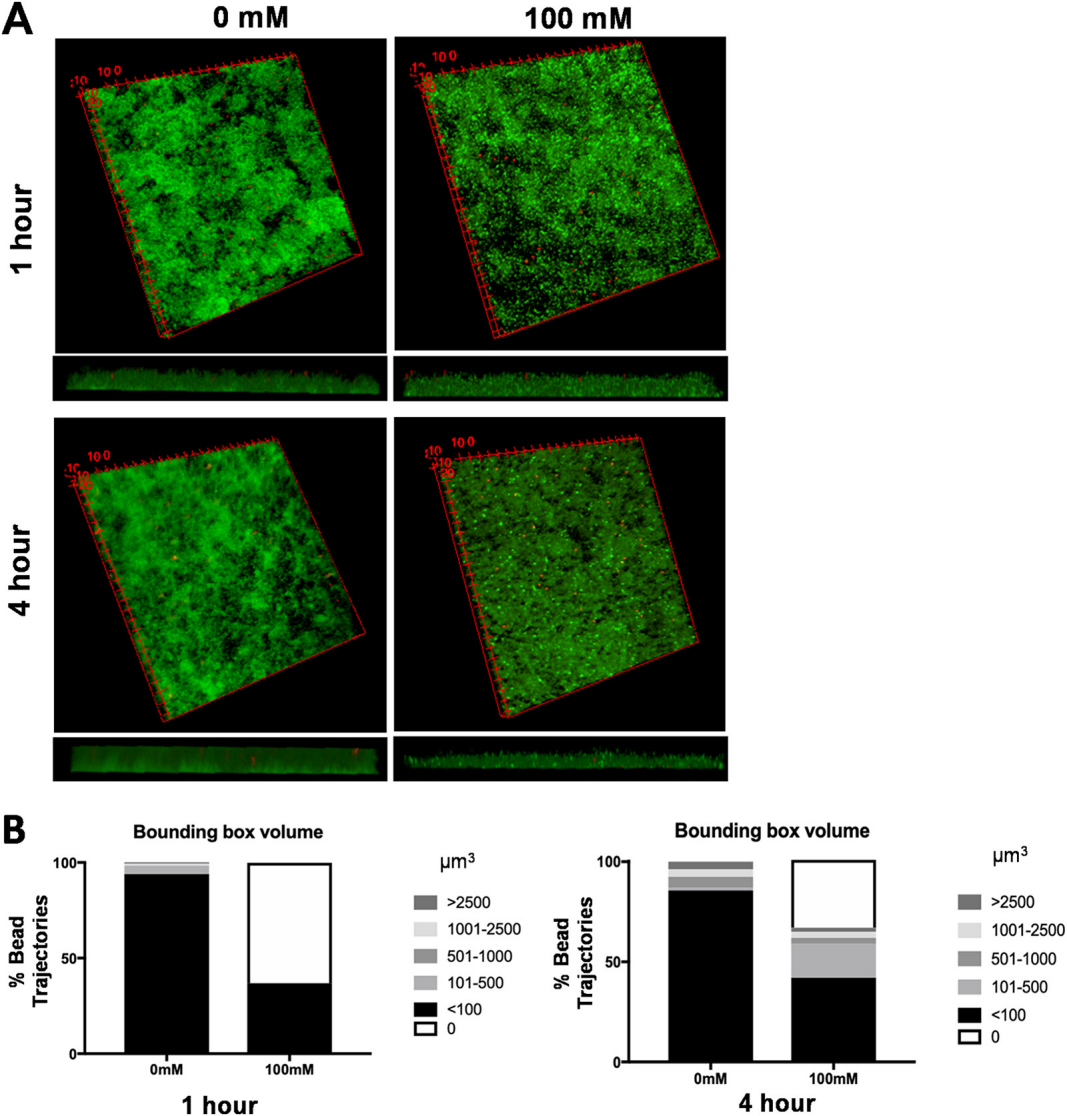
Addition of nitrate leads to disruption of biofilm integrity in the mature biofilm. (A) Wild-type *S.* Typhimurium biofilms were grown on round coverslips for 72 h at 28°C. The mature biofilms were washed and treated with 100 mM NaNO_3_ for 1 to 4 h and stained with SYTO 9 (green). Red fluorescently labeled glyoxylate beads were added to the biofilm and imaged using confocal microscopy (63× oil magnification). Shown are 3D views created using ImageJ. (B) Percentage of bead trajectories in indicated bounding-box volumes (μm^3^) in untreated and NaNO_3_-treated biofilms.

Recently, an assay was designed to characterize biofilm properties on a microscale using bead movement (45). Briefly, small fluorescently labeled glyoxylate beads are added to biofilms, and their movement is tracked using laser-scanning confocal microscopy. Software was developed to analyze the trajectory life span and biofilm density around each bead. There is less bead movement throughout a biofilm when its structure is more compact and rigid, typical of biofilms formed by wild-type *S.* Typhimurium. In contrast, beads penetrate and move through biofilms lacking a compact and rigid structure, such as those formed by E. faecalis and the Salmonella *csgBA* curli mutant (45). We used this bead movement assay to assess if the loose structure observed by 3D imaging of nitrate-treated biofilms was correlated with a loss of biofilm integrity. Mature biofilms were grown as previously described and treated with 0 mM or 100 mM NaNO_3_ for 1 to 4 h, fluorescently labeled glyoxylate beads were added, and samples were imaged using confocal microscopy. There was little to no bead movement within the 0 mM biofilm (Fig. 5A). These biofilms were further analyzed using the ImageJ plugin Mosaic to compute the bead locations and their recorded trajectory data, and trajectory lengths and movement of the beads in the biofilm over time were plotted as surface-covered or bounding-box volumes. In the untreated biofilms, 93% of the trajectories had bounding-box volumes of less than 100 μm^3^ (Fig. 5B), indicating that the biofilm is compact and more rigid. In the biofilms treated with 100 mM NaNO_3_, 42% of the trajectories had bounding-box volumes of less than 100 μm^3^, and 17% of the trajectories had bounding-box volumes of 101 to 500 μm^3^. Interestingly, 34% of the trajectories had bounding-box volumes of 0 μm^3^ (Fig. 5B). A bounding-box volume of 0 μm^3^ indicates either no bead movement or the beads falling completely through the biofilm to rest at the glass coverslip interface. Biofilms left untreated did not have any trajectories with a bounding-box volume of 0 μm^3^. Taken together, these data show that nitrate can alter the structure of a mature biofilm, decreasing its compactness and rigidity and compromising its integrity. We hypothesize this loss of integrity results when Salmonella organisms switch to a more motile phenotype to chemotax toward higher levels of nitrate, the same behavior seen *in vivo* in the inflamed intestine during infection.

### Inhibition of nitrate production in the gut during infection increases *csgD* expression and curli production *in vivo*.

**FIG 6:**
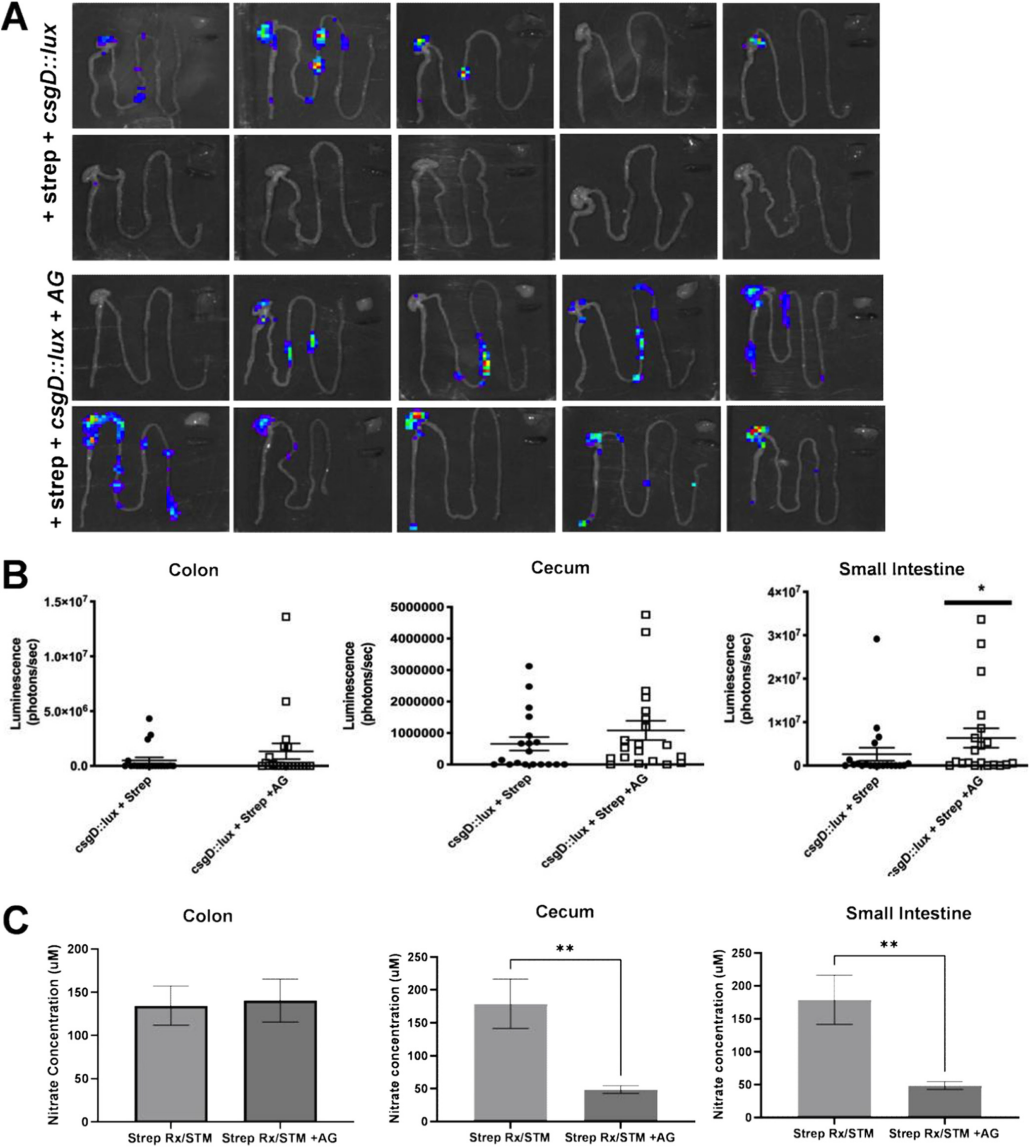
Inhibition of nitrate in the gut during oral infection increases *csgD* expression and curli production. (A) Female C57BL/6 mice (6 to 8 weeks of age) were pretreated with streptomycin and then inoculated orally with 10^8^ CFU of *S.* Typhimurium *csgD*::*luxCDABE* strain. One group of mice was given AG in their drinking water *ad libitum*. Animals were euthanized 96 h postinfection. *csgD* expression in the intestinal tract, spleen, and liver were measured as light production using an IVIS spectrum imaging system (Perkin Elmer). Each panel shows organs from a different mouse. (B) Luminescence in colon, cecum, and small intestine of individual mice left untreated or treated with AG quantified using Aura. *, *P* < 0.05. (C) Nitrate concentration in the colon, cecum, and small intestine of mice with and without the AG inhibitor. **, *P* < 0.01.

Constitutive inducible nitric oxide synthesis (iNOS) is a source of host-derived nitrate in the murine intestinal tract (46, 47). Oral streptomycin pretreatment of mice prior to inoculation with *S.* Typhimurium increases the expression of *iNOS*, which in turn increases the levels of luminal nitrate (32, 47, 48). To determine whether changes in nitrate in the intestinal lumen alter curli production *in vivo*, we utilized the well-established streptomycin-pretreated mouse model and the iNOS inhibitor aminoguanidine hydrochloride (AG), which decreases nitrate levels when given in drinking water (32, 47, 48). Expression of *csgD*, encoding the curli transcriptional activator (49, 50), was monitored in a group of 20 streptomycin-pretreated C57BL/6 mice after oral inoculation with a wild-type *S.* Typhimurium strain that contains a *csgD* luciferase reporter. At 96 h postinfection, the gastrointestinal tract, spleen, and liver were removed, and light production was monitored. We saw a luciferase signal indicative of *csgD* expression in the intestinal tracts of 14 of 20 mice. However, when luminal nitrate was depleted using the iNOS inhibitor AG chloride, 19 of 20 mice expressed *csgD* in the intestinal tract. No luciferase expression was detected for either group in the livers or spleens (Fig. 6A). When the signal intensity was quantified, the gastrointestinal tract was analyzed in three different sections: the colon, cecum, and small intestine. Although *csgD* expression was detected in all AG-treated mice compared to those that were not AG treated, there was only a significant difference in the small intestine (Fig. 6B). Next, we assessed the concentrations of luminal nitrate in the colon, cecum, and small intestine using a Griess assay as described previously (47). Briefly, after euthanization, the colon, cecum, and small intestine were harvested and cut open to expose the inner mucosal layer. The mucus was gently scraped away from the surface and prepared for analysis. Nitrate was detected in the colon, cecum, and small intestine of all infected mice despite the iNOS inhibitor. There was a significant decrease in the levels of nitrate within the cecum and small intestine of AG-treated mice compared to the nontreated mice (Fig. 6C). It is important to note that although the highest pathology is seen in the cecum in mice, in humans, *S.* Typhimurium invades the ileum in small intestine. Therefore, the mechanism for nitrate regulation of biofilm to motile lifestyle may be important where the bacteria need to establish an infection.

## DISCUSSION

Salmonella enterica serovar Typhimurium is an important enteric pathogen that causes foodborne infections in both animals and humans. The ability of *S.* Typhimurium to thrive in the inflamed gut is crucial for its survival and transmission to a new host. *S.* Typhimurium is present as two populations in the intestinal tract: multicellular biofilm aggregates, which contain curli and are adapted for persistence, and planktonic cells that are motile and adapted for virulence (2, 7, 9). The presence of both biofilm formers and planktonic *S.* Typhimurium in the gut suggests that regulatory mechanisms fine-tune its virulence. *S.* Typhimurium utilizes the metabolites generated during intestinal inflammation, including the host-derived nitrate, for anaerobic respiration and expansion (48). However, the environmental triggers for *S.* Typhimurium biofilm production and the switch to motile forms are not known. In this study, we showed for the first time that Salmonella-produced biofilm integrity is regulated in response to host-derived nitrate generated during intestinal inflammation.

**FIG 7:**
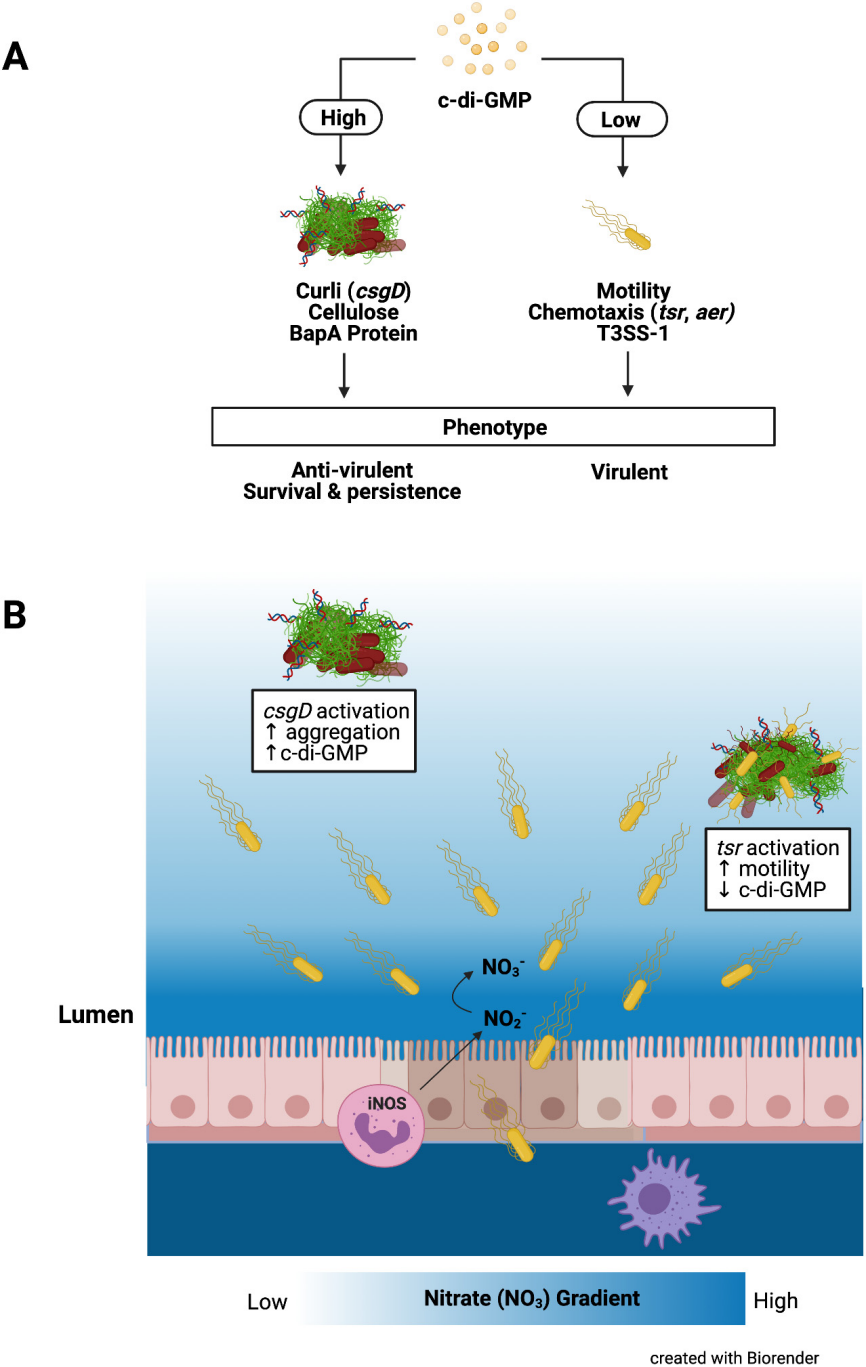
Enteric bacteria regulate biofilm expression in the environment and the intestinal tract in response to c-di-GMP levels. (A) c-di-GMP inversely regulates biofilm formation and motility with high levels of c-di-GMP activating the master regulator, CsgD, increasing the expression and synthesis of extracellular matrix components like curli, cellulose, and BapA and an antivirulent/biofilm phenotype. Low levels of c-di-GMP increase motility and the chemotaxis genes, *tsr* and *aer*, and increase the activation of the T3SS-1 for a more invasive/virulent phenotype. (B) During *S.* Typhimurium infection, planktonic bacteria use their T3SS to invade epithelial cells and induce inflammation, creating a proinflammatory environment. Nitric oxide is release by epithelial cells and activated macrophages and neutrophils, leading to an abundance of nitrate, creating a gradient. Nitrate acts as a cue for *S.* Typhimurium in the outermost region of the biofilm to chemotax toward areas with the greatest nitrate and inflammation, allowing them to invade and disseminate.

Nitrate has a profound effect on *S.* Typhimurium biofilms. We found that biofilms exposed to nitrate lose their integrity, instigating the dispersal of biofilm-associated bacteria. Furthermore, nitrate exposure facilitated a reduction in *csgA* gene expression. There was less CsgA protein expression and production in biofilms treated with nitrate than biofilms grown in the absence of nitrate. In contrast, flagellar motility was increased in the presence of nitrate. c-di-GMP is a second messenger important in signal transduction in a wide variety of bacteria that has been shown to regulate biofilm formation, motility, virulence, the cell cycle, differentiation, and other processes (15, 17, 19, 20, 51, 52). In *Salmonella* biofilms, c-di-GMP inversely regulates biofilm formation and motility. High levels of c-di-GMP are associated with attachment to surfaces, extracellular matrix production, and biofilm formation and repression of motility, whereas low levels of c-di-GMP are associated with motility and virulence (Fig. 7A). We discovered that the addition of nitrate to *S.* Typhimurium cultured under biofilm-forming conditions leads to a dramatic decrease in c-di-GMP levels. These decreases in c-di-GMP levels led to decreased production of biofilm components and increased motility. Overall, these observations support the hypothesis that increasing concentrations of nitrate influence an intracellular molecular switch in c-di-GMP production, and, consequently, the bacterial community shifts from a sessile to a more motile state (43, 51, 53).

*S.* Typhimurium produces curli during acute infections in susceptible mouse strains. We used a streptomycin pretreatment model, in which high levels of nitrate are detected, to evaluate the impact of nitrate on *csgD* expression. Inhibition of mucosal nitrate by AG resulted in increased levels of *csgD* expression, confirming our *in vitro* finding that *S.* Typhimurium responds to changes in luminal nitrate. The nitrate emanating from the mucosal surface of the epithelium is thought to create a gradient of nitrate. It was previously shown that, drawn by nitrate, *S.* Typhimurium uses energy taxis and the methyl-accepting chemotaxis protein Tsr to conduct a flagellum-dependent invasion of Peyer’s patches (30, 47, 54). Our results show that *S.* Typhimurium present in biofilms responds to the same concentration of nitrate, 40 mM, generated during infection. There was a more dramatic response to even 100 mM nitrate, suggesting that the *in vivo* phenotype of *S.* Typhimurium is altered in response to changes in nitrate concentrations. Lower levels of luminal nitrate may dampen chemotaxis, shifting the population toward a less motile, biofilm-producing state.

Like *S.* Typhimurium, other enteric pathogens, as well as human commensal members of *Enterobacteriaceae*, can synthesize curli (25, 38, 39, 55, 56). We hypothesize that enteric bacteria regulate biofilm expression in the environment and the intestinal tract in response to intracellular c-di-GMP levels, which are influenced by environmental cues, such as nitrate concentration (Fig. 7B). The previous report by Smith et al. that the deletion of *narQ* genes in E. coli leads to reduced Congo red binding and curli production is consistent with our idea (57). Furthermore, a recent study showed a unique association between nitrate respiration, biofilm formation, and uropathogenic E. coli pathogenicity (58). Our studies were not performed under conditions where the bacteria can respire nitrate but aimed at dissecting the direct effect of nitrate on the *S.* Typhimurium biofilm. Future studies are needed to delineate the interplay between nitrate respiration and biofilms during infection. However, reported studies suggest that these pathways are conserved in enteric pathogens. For instance, similar to *S.* Typhimurium, excessive environmental nitrate triggers dispersal of sessile Burkholderia pseudomallei from biofilms, leading to an increased abundance of planktonic bacteria. There was a significant reduction of intracellular levels of c-di-GMP grown in the presence of NaNO_3_, indicating that nitrate sensing can control intracellular c-di-GMP levels (59). Although these observations were limited to *in vitro* studies, we expect the regulatory mechanisms for biofilm formation for B. pseudomallei to be similar to those that regulate *S.* Typhimurium biofilm formation, which are the same in culture and in mice. Commensal E. coli cells, as well as pathogenic *S.* Typhimurium, expand their populations in response to gastrointestinal inflammation through nitrate respiration (46, 60–62). The novel role for nitrate in regulation of biofilm integrity proposed here explains how these bacteria rapidly adapt their metabolic needs to fine-tune their motile and sessile populations within the microenvironment by responding to gradients of essential nutrients and metabolites.

In addition to nitrate, other metabolites, such as tetrathionate, fumarate, and succinate, are generated during the host inflammatory response (46, 60–62). These metabolites are also utilized by *S.* Typhimurium as final electron acceptors during respiration. Such metabolites likely create a favorable metabolic niche that provides a growth advantage for *S.* Typhimurium over the competing microbiota and also affect the biofilm in the inflamed gut (63–65). Consistent with this idea, addition of fumarate disturbs the biofilm, similar to what was observed with nitrate (Fig. S6). Overall, our research shows that the metabolic landscape affects the *S.* Typhimurium biofilms in the gut. We envision that concentrations of particular metabolites provide environmental cues as the pathogen travels through the gastrointestinal tract. As biofilms help the pathogen endure the hostile environment of the gut and preformed biofilms could help the bacteria prepare for the external environment, regulation of the sessile to motile switch could be an advantage for the pathogen, enabling establishment of a successful infection and guaranteeing efficient transmission.

10.1128/mBio.02886-21.6 FIG S6 Fumarate significantly decreases biofilm mass. Wild-type *S.* Typhimurium grown in the presence or absence of sodium fumarate for 72 h at 28°C. Biofilms were analyzed using crystal violet staining. ****, *P* < 0.0001. Download FIG S6, TIF file, 0.3 MB. Copyright © 2022 Miller et al. 2022 Miller et al. https://creativecommons.org/licenses/by/4.0/ This content is distributed under the terms of the Creative Commons Attribution 4.0 International license.

## MATERIALS AND METHODS

### Bacterial strains and growth conditions.

Salmonella sp. strain IR715, a fully virulent, spontaneous nalidixic acid-resistant derivative of strain ATCC 14028 (36), was grown in Luria-Bertani (LB) broth supplemented with 50 μg/mL nalidixic acid at 37°C. The IR715 strain was transformed with pMMB-Gm-Bc3-5AAV plasmid carrying a c-di-GMP biosensor (44), kindly provided by Fitnat Yildiz (University of California, Santa Cruz, Santa Cruz, CA). The biosensor works through a tandem riboswitch located upstream of the open reading frame for TurboRFP, which is regulated by c-di-GMP and contains a constitutively active Amcyan cassette, which is used as a plasmid copy number normalizer (66). The reporter strain was grown statically in LB supplemented with 15 μg/mL gentamicin at 37°C unless otherwise noted. The isogenic *csgBA* mutant was previously described (8). The *bscE* mutant derived from the ATCC strain Salmonella enterica serovar Typhimurium 14028 was a gift of John Gunn (The Ohio State University, Columbus OH). *S.* Typhimurium expressing the luciferase plasmid under the control of the *csgD* promoter (P*csgD*::*luxCDABE*) was described previously (67). The strains were grown in LB supplemented with 50 μg/mL nalidixic acid with shaking at 37°C overnight.

### Isolation of biofilm formers and planktonic cell subpopulations.

To isolate the two subpopulations found within the biofilm, wild-type IR715 was grown under biofilm-inducing conditions as previously described (9). Briefly, 500 μL of *S.* Typhimurium overnight culture was added to 150 mL of no salt LB. The culture was grown with shaking at 26°C. To assess the effect of NaNO_3_ on biofilm production, 40 mM or 100 mM NaNO_3_ was added to the culture for an additional 4 to 8 h. Biofilm cultures were transferred to 50-mL conical tubes and subjected to low-speed centrifugation (210 relative centrifugal force for 3 min at 4°C). The supernatant fractions (containing the planktonic subpopulation) and the cell aggregates (containing the biofilm formers) were transferred into 1.5-mL Eppendorf tubes for additional processing. The two subpopulations were pelleted to be used for RNA analysis or Western blotting.

### Crystal violet staining.

To assess the effect of NaNO_3_ on biofilm growth, overnight cultures of *S.* Typhimurium and the isogenic *csgBA* mutant (as a negative control) were diluted in no salt LB broth and grown at 28°C in 0 mM, 10 mM, 25 mM, 50 mM, or 100 mM NaNO_3_. After 72 h, the medium was removed by aspiration, and the biofilm pellicles were washed with sterile PBS (sPBS). After washing, the pellicle was allowed to dry and stained with a 1% crystal violet solution (C581; Fisher). Excess crystal violet was removed, and 33% acetic acid was added to the biofilm before the spectrum from 570 to 595 nm was monitored using a BMG Labtech POLARstar Omega plate reader. The experiment was performed in triplicate. To image the pellicle-associated biofilm on the tube of the static 5-mL cultures, biofilms were stained with 1% crystal violet. Once the liquid culture was removed from the tube, 1% crystal violet was carefully added so that the biofilm was not displaced. Once the pellicle was fully stained, the remaining crystal violet was carefully removed. The crystal violet-stained pellicle-associated biofilm was then imaged using an iPhone 6s.

### Curli and cellulose morphotypes.

Congo red and Coomassie blue indicator plates were supplemented with 0 mM, 40 mM, or 100 mM NaNO_3_. Briefly, 50 μg/mL of Congo red and 10 μg/mL of Coomassie blue were added to 1 liter of yeast extract supplemented with Casamino Acids (YESCA). This solution was divided equally among 250-mL flasks containing a predetermined amount of NaNO_3_, mixed, and poured into 100- by 15-mm petri dishes. Strains were spotted and incubated for 72 h at 28°C. Representative images of the colony morphology of wild-type *S.* Typhimurium, *csgBA* mutant, and *bcsE* mutant were photographed using a magnified colony counter.

### Curli and flagella detection using SDS-PAGE and immunoblotting.

To detect curli synthesis, bacteria were grown on YESCA plates supplemented with 0 mM, 40 mM, or 100 mM NaNO_3_ at 28°C for 72 h. Colonies were recovered from plates, and curli fibrils were depolymerized using HFIP as described previously (68). Next, HFIP-treated extracts were separated by SDS-PAGE, and proteins were transferred to Immobilon-P (Millipore) membranes using a Trans-Blot semidry transfer cell (Bio-Rad). The presence of CsgA was detected using rabbit anti-CsgA serum as described previously (24). To detect flagellin expression, the biofilm-former subpopulation was isolated and separated by SDS-PAGE and transferred to an Immobilon-P membrane using semidry transfer. The presence of flagellin was detected using anti-H serum from BD Difco (1:1,000). Membranes were imaged on the Odyssey imaging system (LI-COR).

### Detection of c-di-GMP.

The *S.* Typhimurium c-di-GMP reporter strain was used to confirm c-di-GMP levels. To optimize conditions for biofilm production, the c-di-GMP reporter strain was grown statically at 28°C in no salt LB with appropriate antibiotic selection supplemented with 0 mM, 40 mM, or 100 mM NaNO_3_. Samples were concentrated 10-fold and spotted onto a microscopy slide. Spots were allowed to dry completely before imaging on a Leica SP5 microscope with a TCS confocal system using a sequential scan at 100× oil magnification. Images were taken from a minimum of 10 fields for each condition. Amcyan- and TurboRFP-positive cells were enumerated using ImageJ. Thresholded images of Amcyan-positive and TurboRFP-positive cells were counted using the “Analyze particles” function of ImageJ. The percentage of c-di-GMP-positive cells was determined as the number of TurboRFP-positive cells divided by the number of Amcyan-positive cells for each individual field times 100. The final mean percentage for each condition was determined by averaging the percentages of each individual field.

### Motility assay.

For motility assays, plates containing 10 g/liter tryptone, 5 g/liter NaCl, and 0.3% agar were supplemented with 0 mM, 40 mM, or 100 mM NaNO_3_. Plates were inoculated with a single colony from an agar plate using a 100-μL pipette tip and incubated at 28°C for 15 h. The diameter of each halo was measured. These experiments were performed in triplicate.

### Real-time PCR.

RNA was extracted, by following the manufacturer’s protocol (TRIzol Max bacterial RNA isolation kit), from biofilm-forming and planktonic cell subpopulations. Briefly, bacterial cells were spun down and treated with Max bacterial enhancement reagent before RNA was extracted with 1 mL TriReagent. Reverse transcription of total RNA (1 μg) was performed in a 50-μL volume according to the manufacturer's instructions (TaqMan reverse transcription reagents; Applied Biosystems). Real-time PCR was performed using the SYBR green method (Applied Biosystems) according to the manufacturer's instructions. Real-time PCR was performed for each cDNA sample (5 μL per reaction mixture) in duplicate using the Thermo Fisher 7900HT fast real-time PCR system. Primers for *csgA* and 16S rRNA were used. Results were analyzed using the comparative threshold cycle method with normalizing to 16S rRNA. Fold increases in *csgA*-expressing bacteria were calculated relative to the average level in the *csgBA* mutant.

### Liquid-interface coverslip assay.

Bacterial cultures were analyzed for biofilm formation as previously described (69), with a few modifications. *S.* Typhimurium was grown overnight at 37°C with shaking and then diluted 1:100 in no salt LB. Diluted cultures were added to wells of a flat-bottom 24-well plate, and sterile glass coverslips were inserted into each well. The plate was tilted at an angle until the meniscus sat along the midline of the coverslip. After incubation at 28°C for 72 h, the medium was removed and coverslips were gently washed with sPBS and stained with SYTO 9 green fluorescent nucleic acid stain (Invitrogen). Samples were incubated with no salt LB supplemented with 0 mM, 40 mM, or 100 mM NaNO_3_ for 1 h or 4 h before washing. Supernatant was collected at 1 h and 4 h for bacterial enumeration. Biofilms were stained with SYTO 9 green fluorescent nucleic acid stain (Invitrogen) and washed before coverslips were placed onto clean multitest well slides, biofilm side down. Slides were imaged on a TCS confocal system using a Z stack and 63× oil magnification.

### Biofilm bead movement and penetrance assay.

Bacteria were grown and stained as described in the paragraph above; however, after incubation with NaNO_3_, 1 mL of fluorescently labeled glyoxylate beads was added. After 1 min, the biofilm was washed carefully, and the coverslip was placed onto a clean glass slide, biofilm side down. Slides were imaged on a TCS confocal system using 63× oil magnification. Bead movement and trajectories were analyzed as described previously using VRL Studio (45, 70). Trajectories were computed using the Particle Tracker 2D/3D Mosaic plug-in. The trajectories were analyzed on VRL Studio to determine trajectory lengths and bounding-box volumes.

### *In vivo* infection of mice.

Female C57BL/6 mice were purchased from Jackson Laboratories. The streptomycin-pretreated mouse model was described previously (71, 72). Briefly, 6- to 8-week-old mice were inoculated intragastrically with 20 mg of streptomycin (0.1 mL of a 200-mg mL^−1^ solution in water) 24 h before bacterial inoculation. Bacteria were grown with shaking in LB broth supplemented with kanamycin at 37°C overnight. Mice were inoculated intragastrically with 0.1 mL of 10^7^ to 10^8^ CFU of *S.* Typhimurium IR715 containing the luciferase plasmid. If appropriate, aminoguanidine chloride (AG) was added to the drinking water at a concentration of 1 mg/mL starting on the day of the inoculation and for the remainder of the experiment (47, 61). Animals were euthanized 96 h postinfection. Luciferase signal in the intestinal tract, spleen, and liver was measured using an IVIS Spectrum imaging system (Perkin Elmer). Images were analyzed using Aura imaging software (Spectral Instruments Imaging). Nitrate concentration within the small intestine, cecum, and colon was measured using a Griess assay (Promega) by following the manufacturer’s protocol, with modifications (47). Briefly, the mucus layer of the cecum, colon, and small intestine was gently removed and collected in an Eppendorf tube containing 200 μL of sPBS on ice. Samples were thoroughly homogenized and centrifuged to remove any remaining debris. Supernatants were collected and 50 μL was placed in a 96-well plate with a mixture containing Griess reagent I, Griess reagent II, HCl (1 M), vanadium III chloride (0.2 mM), and deionized water. Absorbance was measured at 540 nm. A standard curve was generated, and a line of best fit was used to determine the nitrate concentrations based on the absorbance values.
