# Supplementary figures and images for: Nitrate Is an Environmental Cue in the Gut for Salmonella enterica Serovar Typhimurium Biofilm Dispersal through Curli Repression and Flagellum Activation via Cyclic-di-GMP Signaling
Source: mBio. 2022 Feb 8;13(1):e02886-21. doi: 10.1128/mbio.02886-21 (PMC8822344; doi:10.1128/mbio.02886-21)

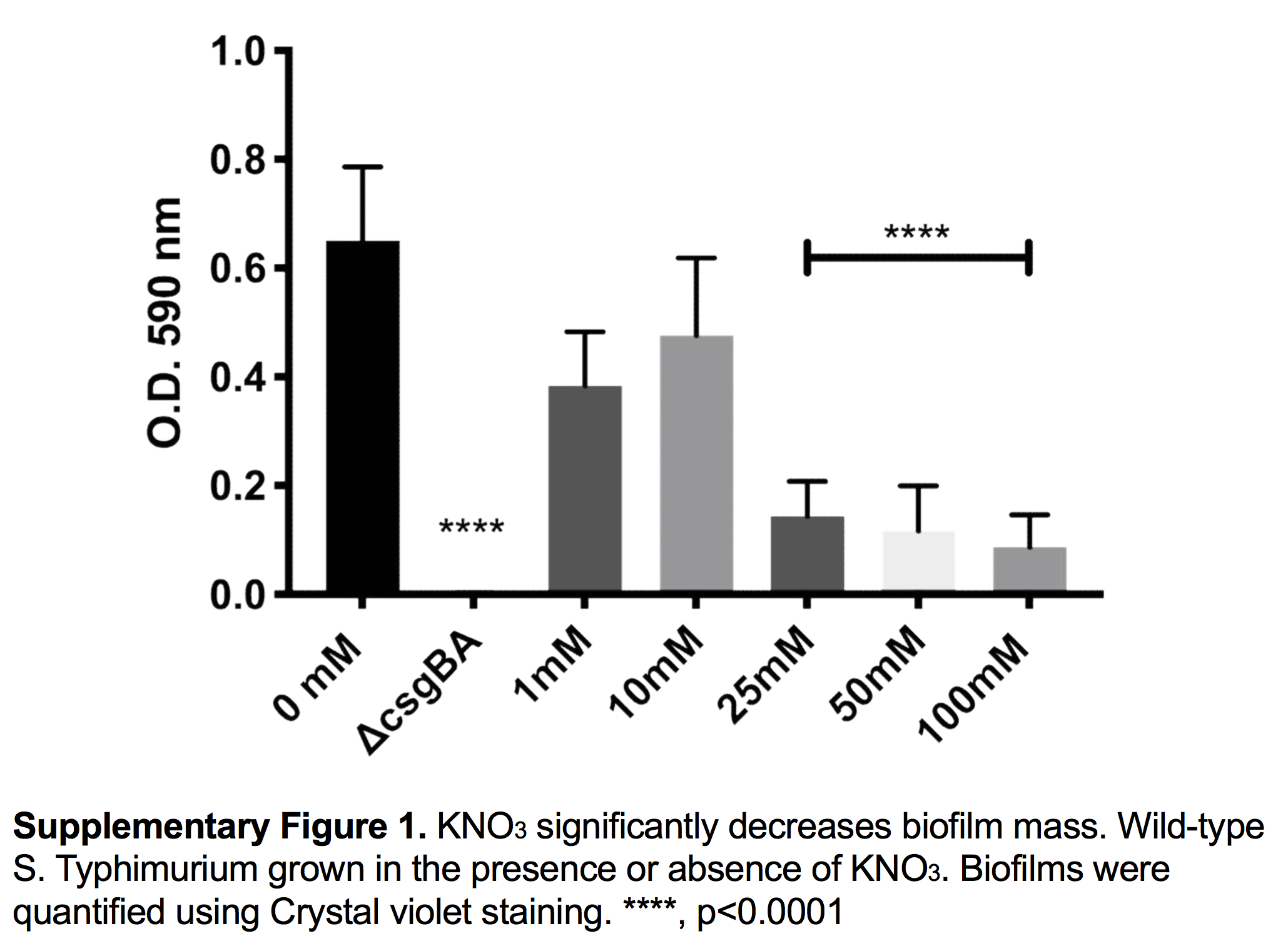

Supplement: FIG S1 [file mbio.02886-21-sf001.tif]

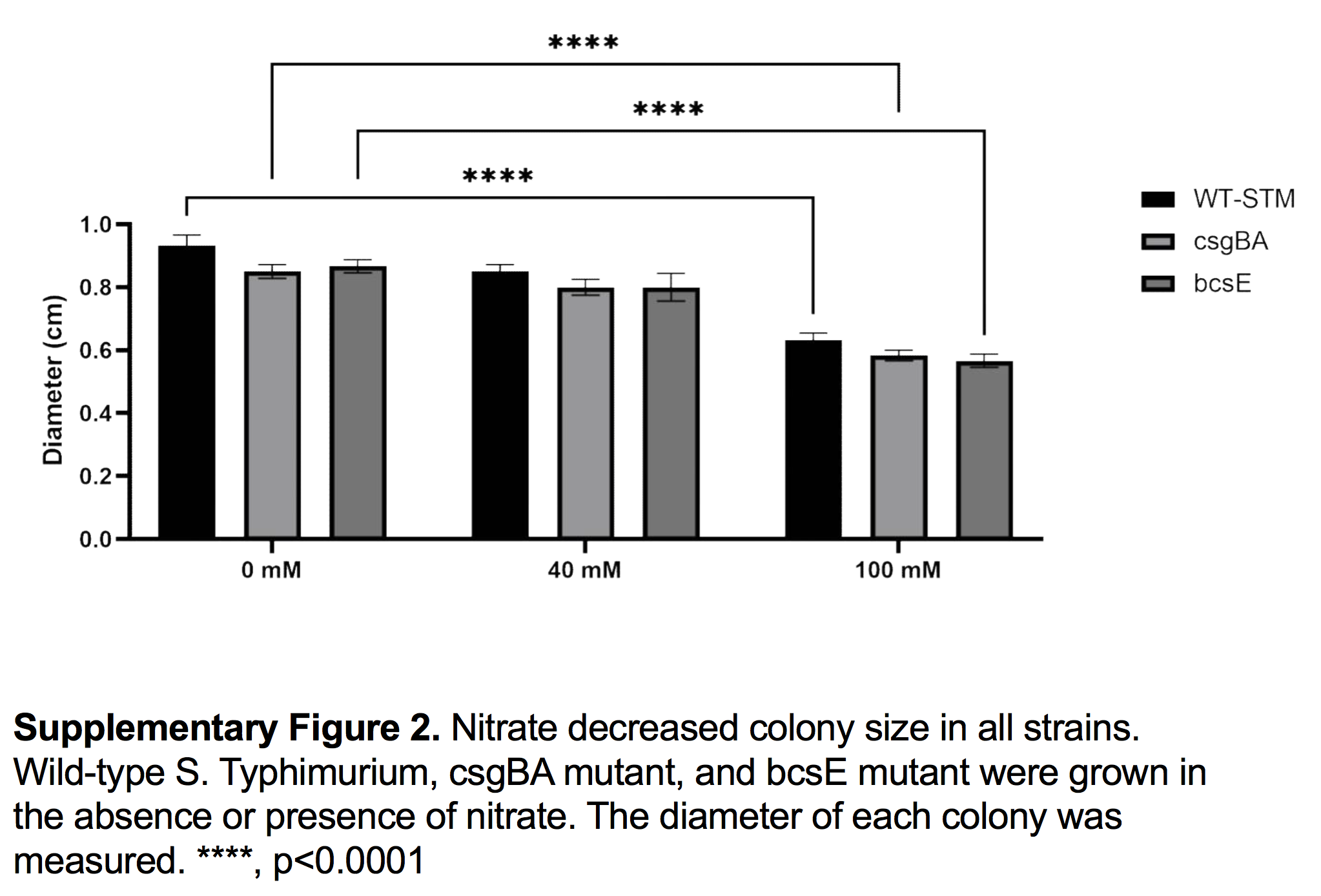

Supplement: FIG S2 [file mbio.02886-21-sf002.tif]

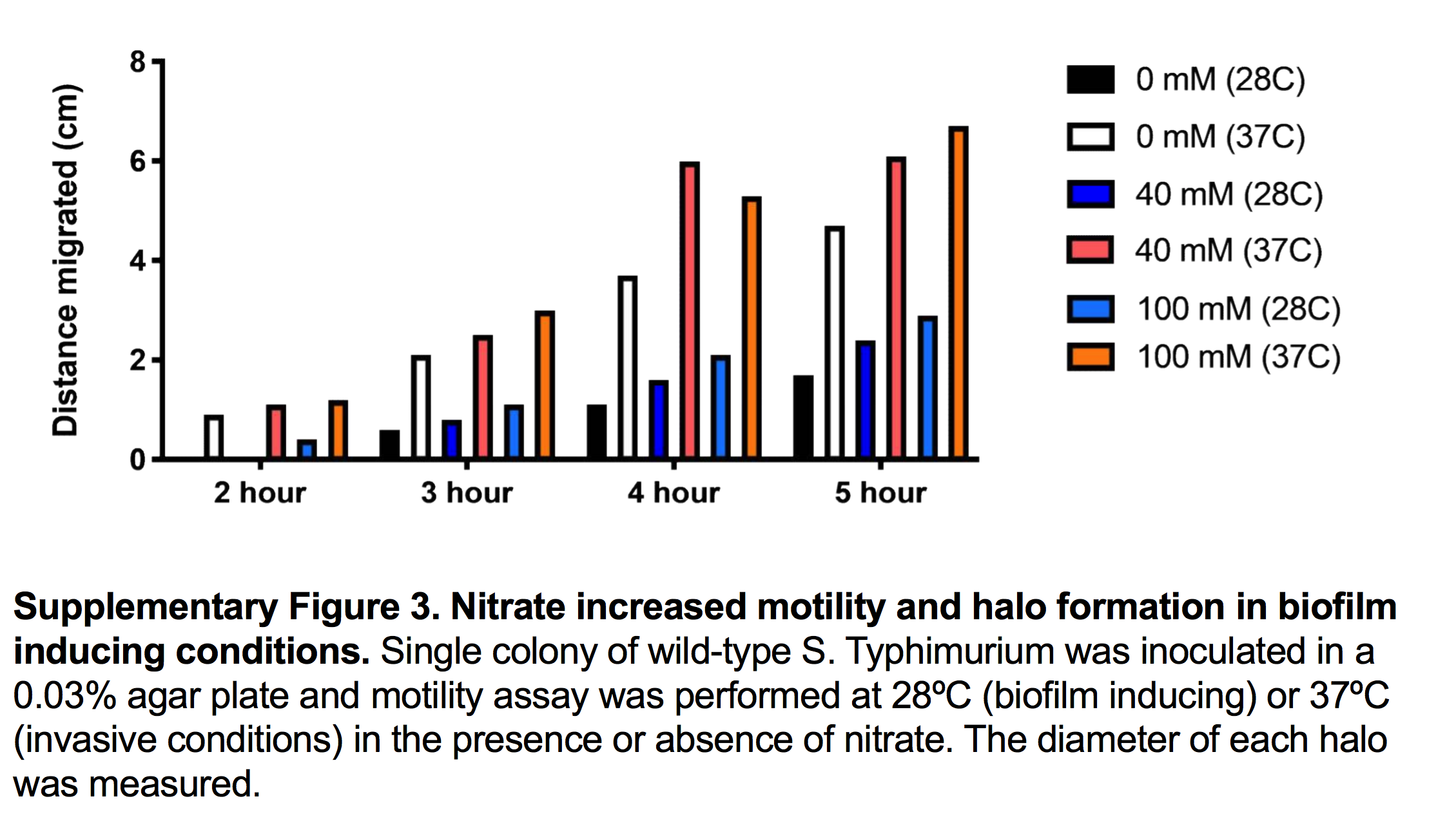

Supplement: FIG S3 [file mbio.02886-21-sf003.tif]

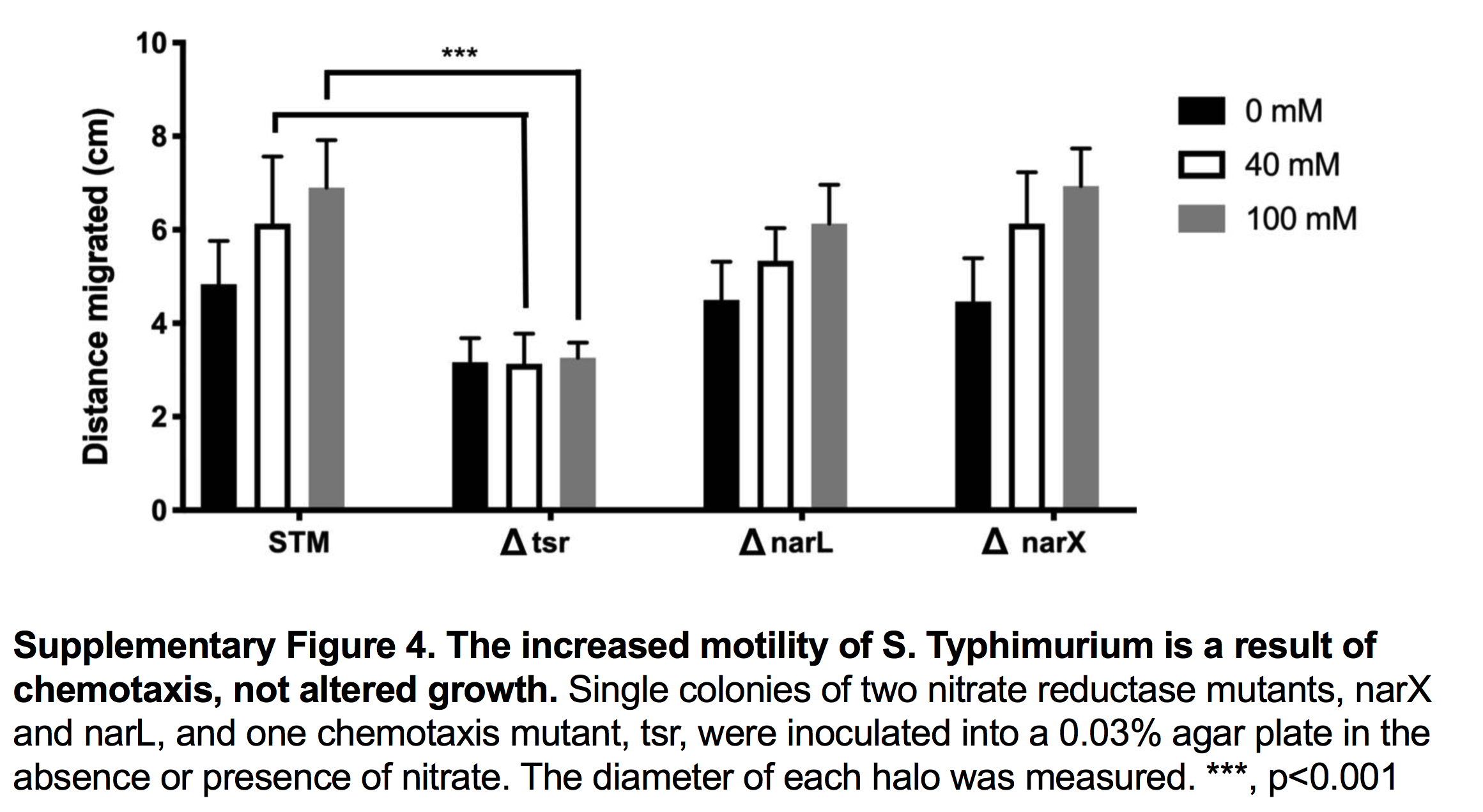

Supplement: FIG S4 [file mbio.02886-21-sf004.tif]

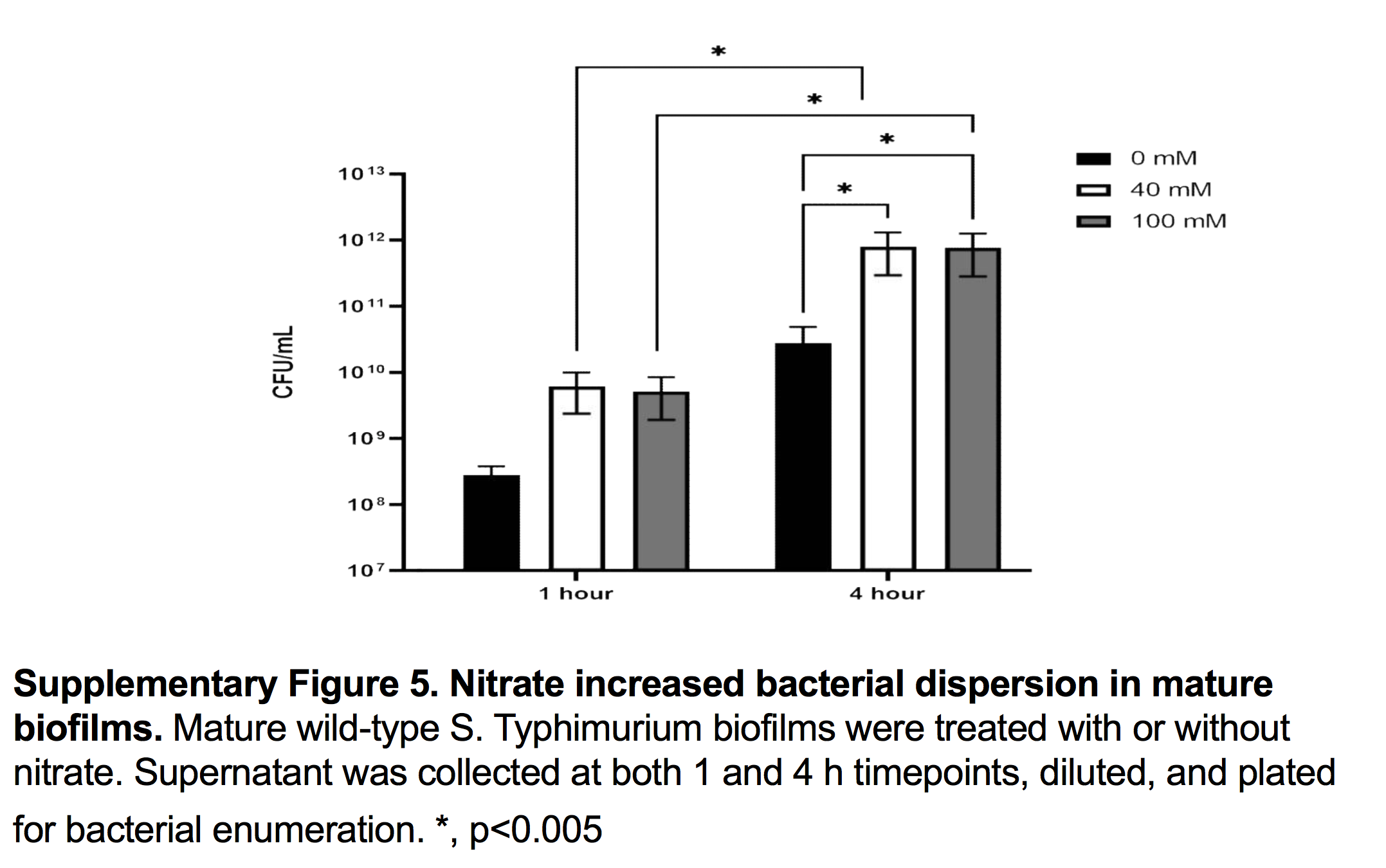

Supplement: FIG S5 [file mbio.02886-21-sf005.tif]

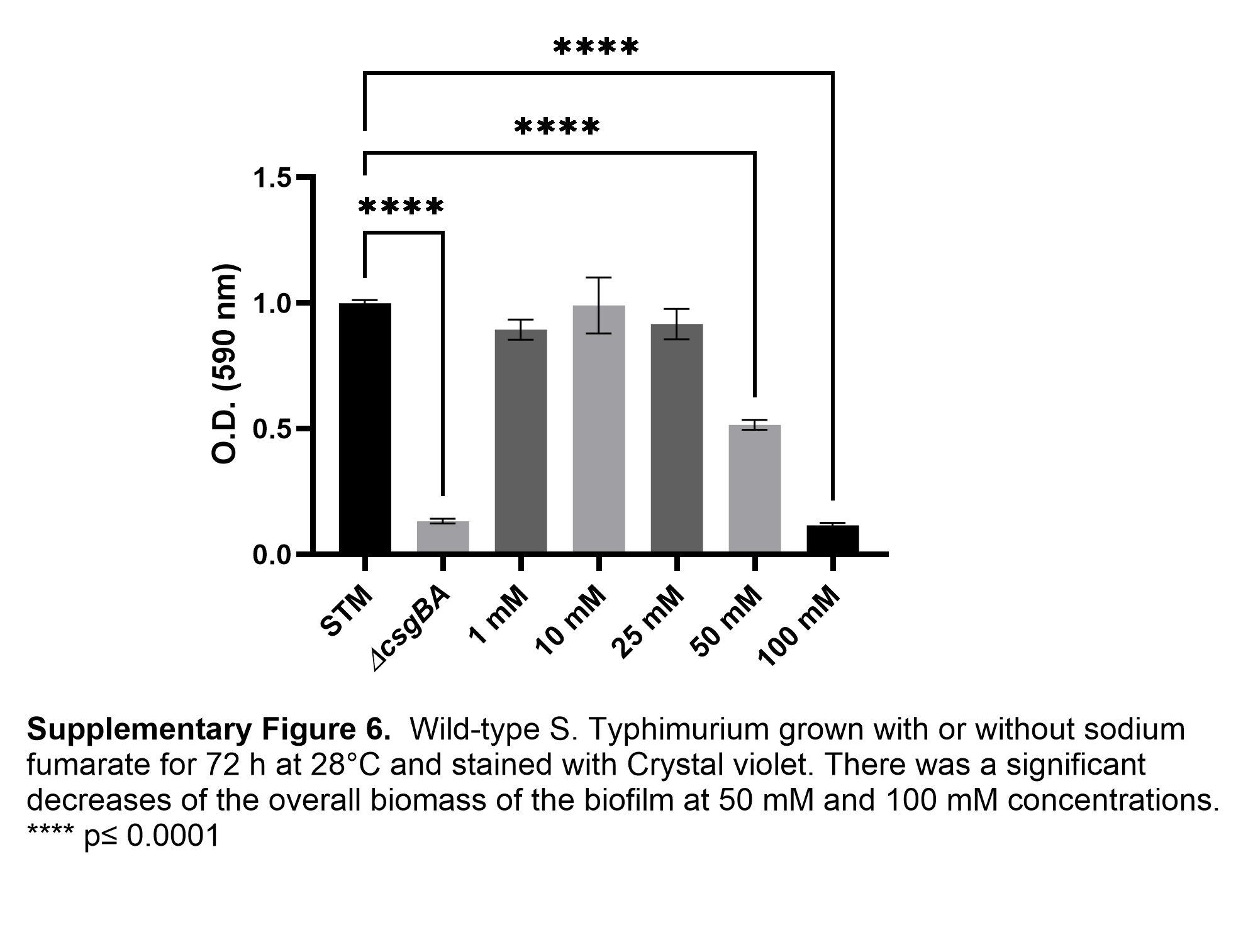

Supplement: FIG S6 [file mbio.02886-21-sf006.tif]
